# Construction and validation of “WCH‐nomogram” for predicting the prognosis after resection of colorectal liver metastases

**DOI:** 10.1002/cam4.7222

**Published:** 2024-05-02

**Authors:** Chuang Jiang, Weixing Liu, Zechuan Jin, Ling Lan, Lin Xu, Ao Du, Song Peng, Yong Zeng, Haichuan Wang, Mingheng Liao, Jin Zhou

**Affiliations:** ^1^ Division of Liver Surgery, Department of General Surgery and Laboratory of Liver Surgery, and State Key Laboratory of Biotherapy and Collaborative Innovation Center of Biotherapy, West China Hospital Sichuan University Chengdu Sichuan China; ^2^ Department of Colorectal Cancer Center, West China Hospital Sichuan University Chengdu Sichuan China

**Keywords:** Colorectal cancer, colorectal liver metastasis, nomogram, survival

## Abstract

**Background:**

The prognostic predictive tool for patients with colorectal liver metastasis (CRLM) is limited and the criteria for administering preoperative neoadjuvant chemotherapy in CRLM patients remain controversial.

**Methods:**

This study enrolled 532 CRLM patients at West China Hospital (WCH) from January 2009 to December 2019. Prognostic factors were identified from the training cohort to construct a WCH‐nomogram and evaluating accuracy in the validation cohort. Receiver operating characteristic (ROC) curve analysis was used to compare the prediction accuracy with other existing prediction tools.

**Results:**

From the analysis of the training cohort, four independent prognostic risk factors, namely tumor marker score, KRAS mutation, primary lymph node metastasis, and tumor burden score were identified on which a WCH‐nomogram was constructed. The C‐index of the two cohorts were 0.674 (95% CI: 0.634–0.713) and 0.655 (95% CI: 0.586–0.723), respectively, which was better than the previously reported predication scores (CRS, m‐CS and GAME score). ROC curves showed AUCs for predicting 1‐, 3‐, and 5‐year overall survival (OS) of 0.758, 0.709, and 0.717 in the training cohort, and 0.860, 0.669, and 0.692 in the validation cohort, respectively. A cutoff value of 114.5 points was obtained for the WCH‐nomogram total score based on the maximum Youden index of the ROC curve of 5‐year OS. Risk stratification showed significantly better prognosis in the low‐risk group, however, the high‐risk group was more likely to benefit from neoadjuvant chemotherapy.

**Conclusions:**

The WCH‐nomogram demonstrates superior prognostic stratification compared to prior scoring systems, effectively identifying CRLM patients who may benefit the most from neoadjuvant chemotherapy.

## Graphical abstract

For the first time, we have integrated tumor marker scoring as one of the prognostic indicators in constructing a predictive model for colorectal liver metastases (CRLM). In comparison to existing predictive models, our model exhibits enhanced predictive accuracy. Our research holds the potential to facilitate individualized survival prediction for CRLM patients and provide guidance to clinicians in making decisions regarding neoadjuvant chemotherapy.

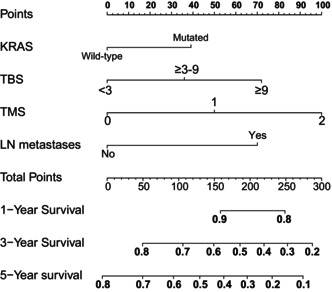

## 1 INTRODUCTION

Colorectal cancer (CRC) is a major global health concern, ranking as the third most common cancer worldwide.^1^, ^2^, ^3^ Nearly one third of CRC patients present with liver metastasis upon initial evaluation, and liver metastasis might be detected in half of CRC patients throughout the disease's progression.^4^, ^5^, ^6^, ^7^, ^8^ However, colorectal cancer liver metastasis (CRLM) holds the potential for a cure, unlike most metastatic cancers.^9^, ^10^ Surgical resection is the most promising treatment for CRLM patients, with patients losing their chance for resection having a 5‐year survival rate of less than 5%.^11^ The prognosis of CRLM has been further enhanced with the development of multidisciplinary treatment, which includes radiotherapy, chemotherapy, and other systematic treatments in addition to surgical intervention. To ensure the best long‐term outcome, it is critical to determine whether neoadjuvant chemotherapy is necessary and the optimal timing for surgery.

Tumor biology is a crucial factor in predicting patient survival and recurrence. Various tools have been developed based on clinicopathological characteristics to predict recurrence risk, assess the timing of surgery, and determine whether neoadjuvant chemotherapy will be beneficial.^12^, ^13^, ^14^, ^15^ The clinical risk score (CRS), proposed by Fong et al. in 1999, is the earliest and most widely used prognostic tool.^15^ The CRS is based on five easy‐to‐obtain clinical indicators: disease‐free interval less than 12 months, preoperative carcinoembryonic antigen (CEA) level >200 ng/mL, largest hepatic metastatic tumor size >5 cm, node‐positive primary, and number of hepatic metastatic tumors >1. Each risk factor should be assigned one point, for a total score of 5 points. Patients are then categorized as low‐risk (0–1 point) or high‐risk (2–5 points). The CRS is widely used worldwide due to its simple calculation method and easily obtainable factors.^16^, ^17^

The significance of genetic and biological factors in predicting the prognosis of CRLM patients has been highlighted in the era of precision medicine. Several genes such as RAS and BRAF have been linked to the therapeutic effect and prognosis of CRLM.^18^, ^19^, ^20^, ^21^, ^22^ Therefore, to improve the accuracy of prediction and guide clinical treatment strategies, some medical centers have added genetic factors to the prediction models. For instance, the genetic and molecular evaluation (GAME) score developed by Margonis et al.^23^ and the modified clinical score (m‐CS) developed by Kristoffer et al.^24^ both exhibit better prediction accuracy than the CRS. Nevertheless, these models have not been widely used and their applicability to diverse patient populations remains to be determined, highlighting the need for further research in this area.

This study aims to address the limitations of previous prognostic models by developing a novel nomogram, referred to as the “WCH‐nomogram,” based on data from our center. The predictive efficacy of the WCH‐nomogram will be compared with the widely used CRS, as well as the GAME score and m‐CS, which incorporate genetic and molecular factors. The unique focus on asian patients with CRLM is significant because there may be distinct differences in genetic and environmental factors that influence the disease course and treatment response. The results of this study may provide valuable insights into the prognostic prediction and decision‐making for neoadjuvant chemotherapy in this population.

## 2 METHODS

### 2.1 Patients and methods

This retrospective study collected clinical and pathological data of patients with colorectal liver metastasis (CRLM) who were admitted to West China Hospital of Sichuan University from January 2009 to December 2019. A total of 532 patients were included in the study cohort, diagnosed by histology and immunohistochemical staining of surgical specimens after hepatectomy. Exclusion criteria: (1) Extrahepatic metastasis before operation; (2) Died within 30 days after operation; (3) Palliative resection (R2 resection) or biopsy only; (4) Emergency operation; (5) Incomplete clinical and pathological data. All patients provided signed informed consent for the operation and agreed to the use of their clinical data for scientific research. This study was approved by the Ethics Committee of West China Hospital under statement number SHEN‐2022‐1455 of September 22, 2022.

### 2.2 Outcomes and data collection

The clinical, pathological and follow‐up data of the included patients were retrospectively collected from the inpatient records and pathological reports. Information collected included: gender, age at diagnosis, basic diseases (hypertension, diabetes, cirrhosis), primary tumor site, primary lymph node metastasis, preoperative neoadjuvant chemotherapy, size and number of liver metastatic lesions, preoperative levels of CEA, carbohydrate antigen 19‐9 (CA19‐9), gene mutation status, tumor differentiation and whether major or minor hepatectomy.

### 2.3 Tumor marker score (TMS)

CEA and CA19‐9 are considered conventional tumor markers associated with the prognosis of CRLM. The X‐tile software is a small single‐function software developed by Yale University that can determine the cut‐point value of continuous variables and facilitate the drawing of survival curves.^25^ X‐tile (version 3.6.1) software was used to determine the optimal cutoff values of CEA and CA19‐9 that affect postoperative OS. Patients were divided into CEA and CA19‐9 low‐level group (0 points) and high‐level group (1 point). The TMS of the patients was given as the total score (CEA score plus CA19‐9 score).

### 2.4 Detection of KRAS mutation

(1) Separate DNA from CRLM surgical specimens; (2) Design primers for KRAS gene fragments; (3) Amplify these regions of interest using polymerase chain reaction (PCR); (4) Detection mutations of KRAS codons 12, 13, and 61 using Sanger sequencing.

### 2.5 Calculation of GAME score and the m‐CS

The GAME score included five indicators,^23^ namely KRAS mutation (1 point), tumor burden score (TBS) (1 or 2 points), lymph node metastasis (1 point), CEA ≥ 20 ng/mL (1 point), extrahepatic metastasis (2 points). The TBS was a prognostic indicator proposed by Sasaki et al. that combined the cumulative impact of tumor size and tumor number on survival.^26^ The calculation formula was TBS^2^ = [maximum lesion diameter]^2^ + [number of tumors]^2^. Patients were divided into three groups according to the TBS value (Group 1: TBS <3 points; Group 2: TBS ≥3–9 points; Group 3: TBS ≥9 points). For m‐CS, three indicators were included,^24^ namely primary lymph node metastasis (1 point), KRAS mutation (1 point) and maximum diameter of liver metastasis >5 cm (1 point).

### 2.6 Follow‐up

Outpatient follow‐up was the main method for patients after surgery, and regular follow‐up was carried out by telephone. The follow‐up plan included the first review 1 month after surgery, reexamination every 3 months in the first 2 years, once every half a year in the third to fifth years, and once a year after 5 years. The follow‐up content included whole abdomen enhanced CT scan or magnetic resonance imaging (MRI), and laboratory examinations such as routine blood testing, tumor markers, liver and kidney function tests, and electrolytes.

### 2.7 Statistical analysis

Statistical analysis was performed using R software (version 3.6.1) and SPSS statistical software (version 23.0, IBM Co., New York, NY). Continuous variables were compared using either the student's *t*‐test or the Wilcoxon signed‐rank test. Categorical variables between groups were compared using either Pearson's *χ* ^2^ test or Fisher's exact test. The Cox proportional hazards regression model was used to identify the independent factors of long‐term prognosis. The univariate analysis was utilized to screen potential prognostic factors, and those with a *p* value of <0.05 were further evaluated in the multivariate analysis, then, the final prognostic factors obtained through univariate and multivariate analysis were included in the construction of the nomogram. The discriminant ability of the nomogram was evaluated using C‐index, AUC, and calibration plots in the training and validation cohort, and compared with CRS, GAME score, and m‐CS. The cutoff value of the nomogram was determined based on the maximum value of the Youden index and was further used for prognostic stratification. We then performed subgroup analyses of the low‐risk and high‐risk groups according to whether or not neoadjuvant chemotherapy was performed. Kaplan–Meier survival curves were analyzed using the log‐rank test.

## 3 RESULTS

### 3.1 Basic clinicopathological characteristics of the study cohort

This study included a total of 532 patients with CRLM who received surgical treatment at West China Hospital of Sichuan University. The median follow‐up time was 43.60 months (IQR 26.35–61.92 months). By the end of the observation period, 253 patients (47.6%) had died and 279 patients (52.4%) had survived. The mean age of the patients was 57.68 years old, with a higher proportion of male patients (66.3%). The overall proportion of patients who received preoperative neoadjuvant chemotherapy was 46.2%. The entire cohort was randomly divided into a training cohort (*n* = 371) and a validation cohort (*n* = 161) with a ratio of 7:3. No significant differences in basic clinicopathological characteristics were observed between the two cohorts. Detailed baseline data of the study cohort are presented in Table 1.

**TABLE 1:** Baseline clinicopathological characteristics of the study cohort.

Variables	Total (*n* = 532, %)	Train cohort (*n* = 371, %)	Validation cohort (*n* = 161, %)	*p* value
Gender				0.463
Male	353 (66.35)	242 (65.23)	111 (68.94)	
Female	179 (33.65)	129 (34.77)	50 (31.06)	
Age (years)				0.910
≤60	301 (56.58)	211 (56.87)	90 (55.90)	
>60	231 (43.42)	160 (43.13)	71 (44.10)	
Diabetes				1.000
No	490 (92.11)	342 (92.18)	148 (91.93)	
Yes	42 (7.89)	29 (7.82)	13 (8.07)	
HBV infection, *n* (%)				0.475
No	509 (95.68)	357 (96.23)	152 (94.41)	
Yes	23 (4.32)	14 (3.77)	9 (5.59)	
Cirrhosis, *n* (%)				1.000
No	520 (97.74)	363 (97.84)	157 (97.52)	
Yes	12 (2.26)	8 (2.16)	4 (2.48)	
Hypertension, *n* (%)				0.526
No	446 (83.83)	314 (84.64)	132 (81.99)	
Yes	86 (16.17)	57 (15.36)	29 (18.01)	
Hepatectomy range, *n* (%)				0.926
Minor	357 (67.11)	248 (66.85)	109 (67.70)	
Major	175 (32.89)	123 (33.15)	52 (32.30)	
Primary site, *n* (%)				0.089
Left colon	413 (77.63)	280 (75.47)	133 (82.61)	
Right colon	119 (22.37)	91 (24.53)	28 (17.39)	
Neoadjuvant, *n* (%)				0.577
No	286 (53.76)	196 (52.83)	90 (55.90)	
Yes	246 (46.24)	175 (47.17)	71 (44.10)	
Metastasis in 12 months, *n* (%)				1.000
No	287 (53.95)	200 (53.91)	87 (54.04)	
Yes	245 (46.05)	171 (46.09)	74 (45.96)	
KRAS status, *n* (%)				0.636
Wild type	344 (64.66)	237 (63.88)	107 (66.46)	
Mutated	188 (35.34)	134 (36.12)	54 (33.54)	
Tumor size, *n* (%)				0.609
≤5 cm	441 (82.89)	305 (82.21)	136 (84.47)	
>5 cm	91 (17.11)	66 (17.79)	25 (15.53)	
Tumor number, *n* (%)				0.397
Single	341 (64.10)	233 (62.80)	108 (67.08)	
Multiple	191 (35.90)	138 (37.20)	53 (32.92)	
Differentiation, *n* (%)				0.891
Poorly	129 (24.25)	88 (23.7)	41 (25.5)	
Moderate	399 (75.00)	280 (75.5)	119 (73.9)	
Well	4 (0.75)	3 (0.8)	1 (0.6)	
TBS, *n* (%)				0.240
<3	192 (36.09)	129 (34.77)	63 (39.13)	
≥3–9	303 (56.95)	212 (57.14)	91 (56.52)	
≥9	37 (6.95)	30 (8.09)	7 (4.35)	
Primary tumor nodal metastases, *n* (%)				1.000
No	207 (38.91)	144 (38.81)	63 (39.13)	
Yes	325 (61.09)	227 (61.19)	98 (60.87)	
CEA, *n* (%)				0.971
≤23.2 ng/mL	391 (73.50)	272 (73.32)	119 (73.91)	
>23.2 ng/mL	141 (26.50)	99 (26.68)	42 (26.09)	
CA19‐9, *n* (%)				0.533
≤32.3 U/mL	267 (50.19)	190 (51.21)	77 (47.83)	
>32.3 U/mL	265 (49.81)	181 (48.79)	84 (52.17)	
TMS, *n* (%)				0.901
0	226 (42.48)	160 (43.13)	66 (40.99)	
1	206 (38.72)	142 (38.27)	64 (39.75)	
2	100 (18.80)	69 (18.60)	31 (19.25)	

Abbreviations: CA19‐9, carbohydrate antigen 199; CEA, carcinoembryonic antigen; KRAS, kirsten rat sarcoma viral oncogene homolog; TBS, tumor burden score; TMS, tumor marker score.

### 3.2 Optimal cutoff values and tumor marker score analysis

**FIGURE 1:**
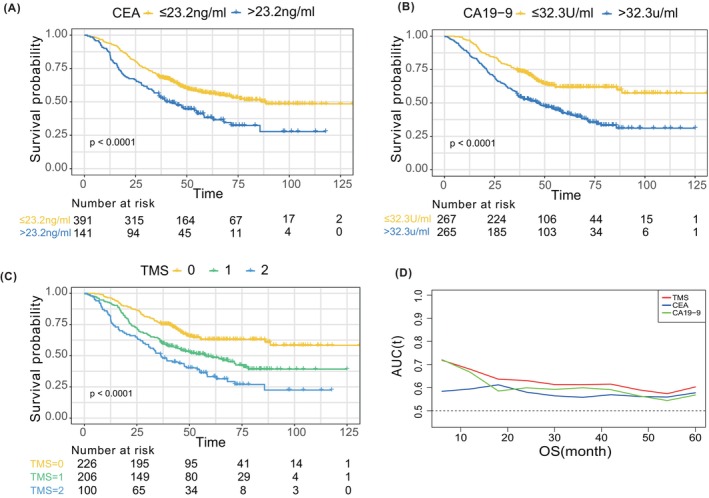
OS of CRLM patients stratified according to CEA, CA19‐9 and TMS, Kaplan–Meier curves grouped by cut‐off value of CEA (A), CA19‐9 (B), and TMS (C). Time‐dependent ROC curves of CEA, CA19‐9, and TMS for predicting overall survival (D). CA19‐9, carbohydrate antigen 199; CEA, carcinoembryonic antigen; CRLM, colorectal liver metastasis; OS, overall survival; TMS, tumor marker score.

The X‐tile software was used to determine the optimal cutoff values of CEA and CA19‐9 for overall survival analysis, which were 23.2 ng/mL and 32.3 U/mL, respectively. Patients with CEA levels less than or equal to 23.2 ng/mL were scored as 0, while those with levels greater than 23.2 ng/mL were scored as 1. Similarly, patients with CA19‐9 levels less than or equal to 32.3 U/mL were scored as 0, while those with levels greater than 32.3 U/mL were scored as 1. The tumor marker score (TMS) was generated by adding the scores of CEA and CA19‐9, which ranged from 0 to 2. The patients were then stratified based on the scores of CEA, CA19‐9, and TMS, respectively. Kaplan–Meier analysis indicated that patients with high CEA or CA19‐9 levels had worse prognoses than those with low CEA or CA19‐9 levels (Figure 1A,B). Moreover, the higher the TMS score, the worse the prognosis (Figure 1C). Time‐dependent ROC curves showed that TMS had the highest AUC value among the three indicators at any time point, indicating that TMS was a better predictor of patient prognosis than CEA or CA19‐9 alone (Figure 1D).

In conclusion, our findings suggest that TMS may be a more appropriate indicator for prognosis. Therefore, TMS was used in subsequent analyses in this study.

### 3.3 Univariate and multivariate analysis of training cohort

The analysis of the training cohort involved both univariate and multivariate methods to assess the prognostic factors for postoperative survival in patients with CRLM. The median overall survival (OS) of the 371 patients who underwent surgery was 45.67 months. Univariate analysis identified four clinicopathological factors—KRAS mutation, TBS, TMS, and positive primary lymph node metastases—as potential risk factors for postoperative survival, with *p*‐values <0.05. Multivariate analysis confirmed that these factors were independent risk factors for postoperative survival, with hazard ratios (HR) of 1.383, 1.356, 1.517, and 1.790, respectively, and *p*‐values <0.001 for TBS, TMS, and primary lymph node metastases, and *p* = 0.035 for KRAS mutation. Notably, primary tumor site, time interval of hepatic metastasis, and preoperative neoadjuvant chemotherapy did not significantly affect postoperative survival (Table 2). Therefore, the study concluded that KRAS mutation, TBS, TMS, and primary lymph node metastases were independent prognostic indicators for patients with CRLM.

**TABLE 2:** Univariate and multivariate analysis of prognostic risk factors in patients with CRLM based on OS.

Variables	Univariate analysis		Multivariate analysis	
	HR (95% CI)	*p* value	HR (95% CI)	*p* value
Gender (male/female)	1.246 (0.924–1.679)	0.149		
Age (>60/≤60 years)	1.171 (0.873–1.571)	0.291		
Diabetes (yes/no)	1.170 (0.700–1.957)	0.549		
HBV infection (yes/no)	1.070 (0.502–2.282)	0.860		
Cirrhosis (yes/No)	0.506 (0.126–2.043)	0.339		
Hypertension (yes/no)	1.291 (0.885–1.883)	0.185		
KRAS status (mutated/wild type)	1.504 (1.116–2.026)	0.007	1.383 (1.023–1.871)	0.035
Neoadjuvant (yes/no)	0.789 (0.588–1.059)	0.114		
Primary site (left/right colon)	0.946 (0.673–1.329)	0.748		
Primary tumor nodal metastases (yes/no)	2.024 (1.466–2.796)	<0.001	1.790 (1.289–2.485)	<0.001
Hepatectomy range (major/minor)	1.268 (0.937–1.715)	0.124		
TBS (<3/≥3–9/≥9)	1.571 (1.239–1.992)	<0.001	1.356 (1.041–1.768)	0.024
Metastasis in 12 months (yes/no)	0.935 (0.697–1.254)	0.652		
TMS (0/1/2)	1.644 (1.360–1.986)	<0.001	1.517 (1.249–1.843)	<0.001
Differentiation (poorly/moderate/well)	0.866 (0.626–1.198)	0.385		

Abbreviations: CRLM, colorectal liver metastasis; KRAS, kirsten rat sarcoma viral oncogene homolog; OS, overall survival; TBS, tumor burden score; TMS, tumor marker score.

**FIGURE 2:**
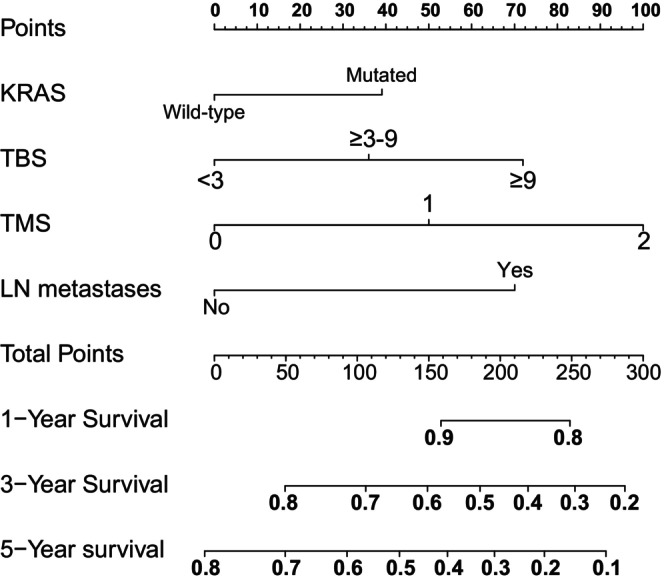
WCH‐nomogram for predicting 1‐, 3‐ and 5‐year overall survival rates of CRLM patients. CRLM, colorectal liver metastasis; KRAS, Kirsten rat sarcoma viral oncogene homolog; LN, lymph node; TBS, tumor burden score; TMS, tumor marker score; WCH, West China Hospital.

**FIGURE 3:**
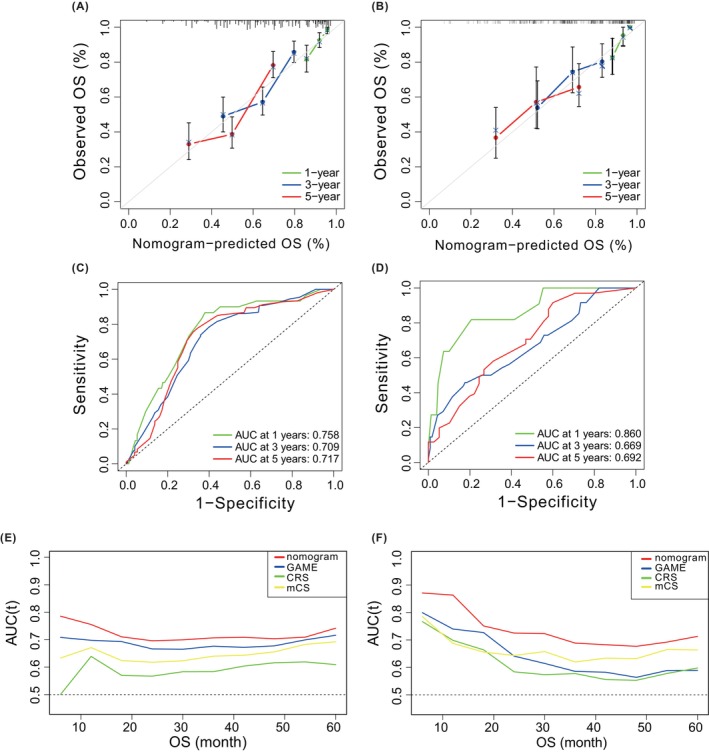
Calibration plots predicting the 1‐, 3‐, and 5‐year OS of patients in the training cohort (A) and validation cohort (B). ROC curves and AUCs of 1‐, 3‐, and 5‐year OS of WCH‐nomogram in training cohort (C) and validation cohort (D). Time‐dependent ROC curves of WCH‐nomogram, CRS, m‐CS, and GAME models for predicting OS of CRLM patients in training cohort (E) and validation cohort (F). CRLM, colorectal liver metastasis; CRS, clinical risk score; GAME, genetic and molecular evaluation; m‐CS, modified clinical score; OS, overall survival; ROC, receiver operating characteristic; WCH, West China Hospital.

The current analysis identified KRAS mutation, TBS, TMS, and primary lymph node as independent prognostic indicators for patients with CRLM. A nomogram called WCH‐nomogram was constructed based on the four important independent risk factors obtained from multivariate analysis to predict the 1‐, 3‐, and 5‐year overall survival rates of patients with CRLM after surgery (Figure 2). The calibration plots of WCH‐nomogram showed a good coincidence between the prediction and actual observation results of OS in 1, 3, and 5 years for both the training and internal verification cohorts (Figure 3A,B). The C‐index values of the training cohort and internal validation cohort were 0.674 (95% CI: 0.634–0.713) and 0.655 (95% CI: 0.586–0.723), respectively, which were higher than the CRS, m‐CS, and GAME scores (0.602 (95% CI: 0.560–0.643), 0.616 (95% CI: 0.576–0.655), 0.642 (95% CI: 0.604–0.681)), respectively. These results indicate that the prognostic accuracy of WCH‐nomogram was the best among the four prediction models. The AUC values of the ROC curves of the 1‐, 3‐, and 5‐year OS for the training cohort and validation cohort were calculated. The AUC values of the training cohort were 0.758, 0.709, and 0.717 (Figure 3C), respectively, and those of the validation cohort were 0.860, 0.669, and 0.692, respectively (Figure 3D).

To compare the power of our WCH‐nomogram with other nomograms, we conducted a comprehensive comparison. The AUC values of 1‐year, 3‐year, and 5‐year OS forecast were generally better than the other three prediction models, with AUC values as follows: CRS (0.669, 0.620, 0.635), m‐CS (0.684, 0.646, 0.661), GAME score (0.713, 0.680, 0.691) (Figure S1; Table 3). Additionally, the Td‐ROC AUC of WCH‐nomogram for predicting prognosis at any month within 5 years after surgery in the training cohort and validation cohort was higher than that of CRS, m‐CS, and GAME score, indicating that WCH‐nomogram had the best prognostic prediction ability (Figure 3D,F).

**TABLE 3:** Comparison of prediction efficiency of the “WCH‐nomogram” with CRS, m‐CS, and GAME score.

Models	C‐index	AUC		
		1 year	3 years	5 years
WCH‐nomogram	0.674	0.758	0.709	0.717
CRS	0.602	0.669	0.620	0.635
m‐CS	0.616	0.684	0.646	0.661
GAME	0.642	0.713	0.680	0.691

Abbreviations: CRS, clinical risk score; GAME, genetic and molecular evaluation; m‐CS, modified clinical score; WCH, West China Hospital.

### 3.4 Risk stratification and prognostic analysis of patients with CRLM

**FIGURE 4:**
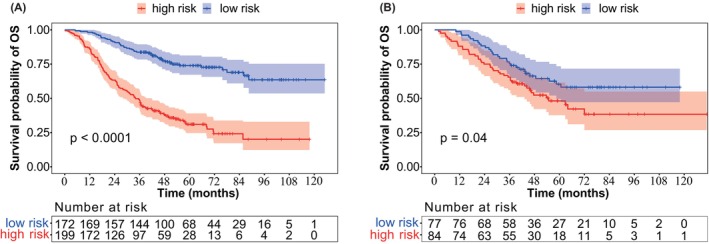
Kaplan–Meier curves of high‐risk and low‐risk group in training cohort (A) and validation cohort (B).

To validate the usefulness of WCH‐nomogram, we calculated the total score of each patient using WCH‐nomogram, and then plotted the ROC curve. The optimal cutoff value was determined based on the maximum value of the Youden index, which was found to be 114.5 points (Figure S2). Consequently, patients were categorized into two groups based on the cut‐off value: high‐risk group (≥114.5) and low‐risk group (<114.5). Kaplan–Meier analysis demonstrated that the survival of patients in the low‐risk group was significantly better than that of those in the high‐risk group in both the training and validation cohorts (Figure 4). These results suggest that WCH‐nomogram is effective for stratifying patients based on distinct prognosis.

### 3.5 Subgroup analysis of neoadjuvant chemotherapy

**FIGURE 5:**
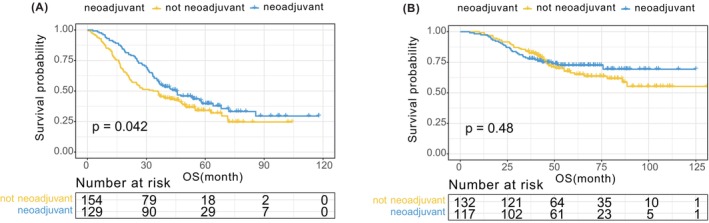
Kaplan–Meier curves of overall survival in the neoadjuvant chemotherapy subgroup of the low‐risk (A) and high‐risk (B) group.

To assess the impact of the WCH‐nomogram on neoadjuvant chemotherapy decision‐making, we conducted further analyses on the low‐risk and high‐risk groups in both cohorts based on whether neoadjuvant chemotherapy was performed. The results demonstrated that neoadjuvant chemotherapy had no significant effect on prognosis in the low‐risk group. However, in the high‐risk group, patients who underwent neoadjuvant chemotherapy had a better prognosis than those who underwent surgery directly (Figure 5). Thus, our analysis suggested that only CRLM patients classified as high‐risk may benefit from neoadjuvant chemotherapy.

To make WCH‐nomogram more convenient for clinical application, we developed an online calculator based on this nomogram (http://crlm.htmlmb.top/). The clinician can obtain the total score of a specific patient by checking the corresponding options, and judge the prognosis and risk of the patient according to the score.

## 4 DISCUSSION

Surgical resection is a highly effective treatment for patients with colorectal liver metastases (CRLM), although a subset of patients may not benefit from this approach. Preoperative neoadjuvant chemotherapy can decrease tumor volume, reducing the extent of resection and surgical risk. However, neoadjuvant chemotherapy carries a risk of tumor progression, leading to the loss of surgical options. Thus, identifying which CRLM patients are likely to benefit from neoadjuvant chemotherapy is critical to optimize medical resource allocation and preserve the opportunity for surgical resection. Therefore, developing screening criteria for neoadjuvant chemotherapy in CRLM patients is important for guiding clinical treatment decisions and improving prognosis.

In the past two decades, clinical risk scores (CRS) have been widely used to guide treatment selection for CRLM patients. However, CRS has several limitations in discriminating patient outcomes. Although CRS has been a valuable tool for choosing treatment options for patients with CRLM in the past two decades, it has several limitations. The AUC of CRS scores, which ranges from 0.53 to 0.68,^27^ is relatively low, and it only relies on laboratory and tumor morphological features, neglecting genetic and biological factors. Both of these factors critically limit its predictive value. As a result, the predictive value of CRS is limited, and various medical centers have constructed new predictive models, such as m‐CS and GAME scores. In this study, we developed a WCH‐nomogram based on four independent prognosis indicators for postoperative survival prediction and neoadjuvant chemotherapy decision‐making in CRLM patients. Our comparison with CRS, GAME, and m‐CS scores demonstrated that the WCH‐nomogram outperformed the other three scoring systems. Furthermore, all four indicators included in the WCH‐nomogram can be obtained before hepatectomy, contributing to the decision‐making of preoperative treatment. Therefore, the WCH‐nomogram has the potential to serve as an additional tool for the management of CRLM patients.

In this study, we utilized TMS as an indicator to evaluate the expression levels of tumor markers CEA and CA19‐9. The preoperative CEA level is influenced by the primary colorectal tumor's characteristics. Various studies have shown that CEA is an independent prognostic factor for CRLM patients.^17^, ^28^ However, the inconsistent cutoff values used in different studies and the failure to consider CA19‐9 as a risk factor in CRS, m‐CS, GAME scores lead to the lack of significance in evaluating the impact of tumor markers on prognosis. Therefore, in this study, we used X‐tile software to determine optimal cutoff values for CEA and CA19‐9 levels. We assigned a score of 0 and 1 to values below and above the optimal cutoff value, respectively. To comprehensively represent the changes in preoperative CEA and CA19‐9 levels' impact on prognosis, we introduced TMS. We believe that this is the first time TMS has been applied to measure prognosis in CRLM patients. Our results indicated that TMS is an independent prognostic factor, with a higher TMS value corresponding to a worse prognosis. The predictive value of TMS was superior to CEA or CA19‐9 alone, potentially improving the prediction efficiency of WCH‐nomogram.

Another important factor in the WCH‐nomogram is the TBS. As early as 2007, a prediction tool called “Metro‐ticket” system was proposed to comprehensively describe the tumor size and number of hepatocellular carcinoma (HCC), which can stratify the prognosis of HCC patients.^29^, ^30^ Ten years later, TBS was proposed as a new “Metro‐ticket” system for an alternative description of tumor size and number in CRLM patients. Kazunari et al.^26^ found that TBS could more scientifically reflect the tumor burden of a particular patient with CRLM than tumor size or number alone. Therefore, we adopted TBS to comprehensively describe the morphological characteristics of liver metastases in this study. Consistent with the previous studies,^23^ our analysis showed that TBS was indeed an important independent risk factor affecting the prognosis of CRLM patients.

In addition to TBS, we identified KRAS mutation as a significant factor in the WCH‐nomogram. KRAS mutation has been increasingly recognized as a strong predictor of overall survival after hepatectomy in CRLM patients. The mutation is associated with a two‐fold increased risk of death and poorer overall and recurrence‐free survival after hepatectomy. The RAS gene is also an important target for colorectal cancer treatment, and KRAS mutation status can predict the therapeutic effect of anti‐epidermal growth factor (EGFR). In a study investigating the combination therapy of the FOLFIRI regimen with cetuximab in colorectal cancer, the findings revealed that, compared to monotherapy, the combination therapy demonstrated significant efficacy improvement in patients with KRAS wild‐type tumors, while no similar benefit was observed in the KRAS mutant population.^31^ Hence, it is suggested that the KRAS gene mutation status holds significance in guiding the decision regarding the inclusion of EGFR monoclonal antibody adjuvant therapy. In addition to KRAS mutations, p53, BRAF, PIK3CA, and MSI status also have an impact on the prognosis of CRLM. The BRAF gene is an oncogene, with the most common mutation being the V600E mutation. The BRAF V600E mutation leads to sustained activation of the RAS/RAF/MAPK signaling pathway. Although its occurrence rate is much lower compared to KRAS mutations, it often indicates a very poor prognosis, and most patients with BRAF mutations often lose the opportunity for surgery.^32^, ^33^ Research on p53 and PIK3CA mutations in CRLM patients is limited. The mutation rate of p53 is relatively high in CRLM, but its impact on prognosis remains unclear. Some studies suggest that it is not significantly associated with the prognosis of CRLM,^34^, ^35^, ^36^ but concurrent mutations in P53 and KRAS may lead to a worse prognosis.^34^ Similarly, PIK3CA mutations can result in sustained abnormal activation of the PI3K‐AKT–mTOR pathway, contributing to the development of CRC. However, the relationship between sole PIK3CA mutations and OS and DFS in CRLM is also controversial, but generally considered to be associated with a poorer prognosis.^37^ Different microsatellite instability (MSI) statuses have varying impacts on the prognosis of CRC. High‐level microsatellite instability (MSI‐H) generally has a better prognosis compared to microsatellite stable (MSS) and low‐level microsatellite instability (MSI‐L) statuses. However, patients with MSI‐H tumors do not benefit from 5‐FU‐based adjuvant chemotherapy but may benefit from immunotherapy.^38^, ^39^ The impact of MSI status on the prognosis of metastatic cancer remains uncertain and warrants further exploration. However, as our study has not yet obtained these mutation statuses, there may be confounding factors such as gene statuses that may affect the accuracy of prognosis assessment. Future prospective clinical studies or inclusion of more patients in research may reduce the influence of these biases.

This study has significant implications for risk stratification of CRLM patients using the nomogram's cutoff values. The high‐risk group had significantly worse overall survival compared to the low‐risk group (*p* < 0.05). We also performed a subgroup analysis based on neoadjuvant chemotherapy and found no significant difference in survival between low‐risk patients who underwent surgery directly and those who received neoadjuvant chemotherapy. However, high‐risk patients benefited from neoadjuvant chemotherapy, and are recommended to receive it before surgery. Low‐risk patients can receive surgical treatment directly to avoid unnecessary chemotherapy. The WCH‐nomogram can aid in developing personalized treatment plans, improve survival outcomes, and save medical costs. To improve external applicability, future studies should verify the reliability of the nomogram through prospective, multicenter research.

Although the construction of nomogram has been verified to have good predictive ability, our study still has some limitations. Firstly, since this study was a retrospective study, the absence of certain information such as BRAF/P53/PIK3CA and other gene mutation statuses, specific chemotherapy regimens and cycles for patients receiving neoadjuvant chemotherapy, along with other unknown confounding factors, may introduce biases into the results. Secondly, since the data is sourced from a single center with a relatively small sample size, external validation is necessary to further assess the model's generalizability. Finally, we excluded some patients due to incomplete clinical data, which may also lead to selection bias.

## 5 CONCLUSIONS

This study has developed a WCH‐nomogram using TMS, TBS, and other clinically important indicators to predict the survival of CRLM patients. The nomogram demonstrates a stronger predictive ability than previously used scoring systems such as CRS, m‐CS, and GAME scores, making it a promising tool for preoperative staging. The WCH‐nomogram can aid in decision‐making regarding the use of neoadjuvant chemotherapy and provide valuable support for personalized treatment planning.

## AUTHOR CONTRIBUTIONS

**Chuang Jiang:** Data curation (lead); supervision (equal); writing – original draft (lead). **Weixing Liu:** Data curation (supporting); methodology (lead); supervision (equal). **Zechuan Jin:** Formal analysis (lead); methodology (supporting); supervision (equal). **Ling Lan:** Data curation (supporting); supervision (equal). **Lin Xu:** Formal analysis (supporting); methodology (supporting). **Ao Du:** Formal analysis (supporting); supervision (equal). **Song Peng:** Data curation (supporting); supervision (equal). **Yong Zeng:** Project administration (equal); supervision (equal); writing – review and editing (equal). **Haichuan Wang:** Project administration (supporting); supervision (lead); writing – review and editing (lead). **Mingheng Liao:** Project administration (lead); supervision (supporting); writing – review and editing (supporting). **Jin Zhou:** Project administration (lead); supervision (equal).

## FUNDING INFORMATION

This work was supported by grants from the National multidisciplinary collaborative diagnosis and treatment capacity building project for major diseases (TJZ202104), the Natural Science Foundation of China (82173248, 82272685, 82002967, and 82202260), the Postdoctoral Science the fellowship of China National Postdoctoral Program for Innative Talents (BX20200225), the Project funded by China Postdoctoral Science Foundation (2022TQ0221), the Science and Technology Major Program of Sichuan Province (2022ZDZX0019), the Sichuan Science and Technology Program (2023YFS0128, 2023NSFSC1874, and 2021YJ0420), 1.3.5 project for disciplines of excellence, West China Hospital, Sichuan University (ZYJC18008, ZYGD22006), the Sichuan University postdoctoral interdisciplinary Innovation Fund (10822041A2103).

## CONFLICT OF INTEREST STATEMENT

There is no conflict of interest.

## ETHICS STATEMENT

This study was approved by the Ethics Committee of West China Hospital of Sichuan University.

## Supporting information


Figure S1.

Figure S2.

## Data Availability

All data included in this study are available upon request by contact with the corresponding author.
