# Supplementary figures and images for: Construction and validation of “WCH‐nomogram” for predicting the prognosis after resection of colorectal liver metastases
Source: Cancer Med. 2024 May 2;13(9):e7222. doi: 10.1002/cam4.7222 (PMC11066479; doi:10.1002/cam4.7222)

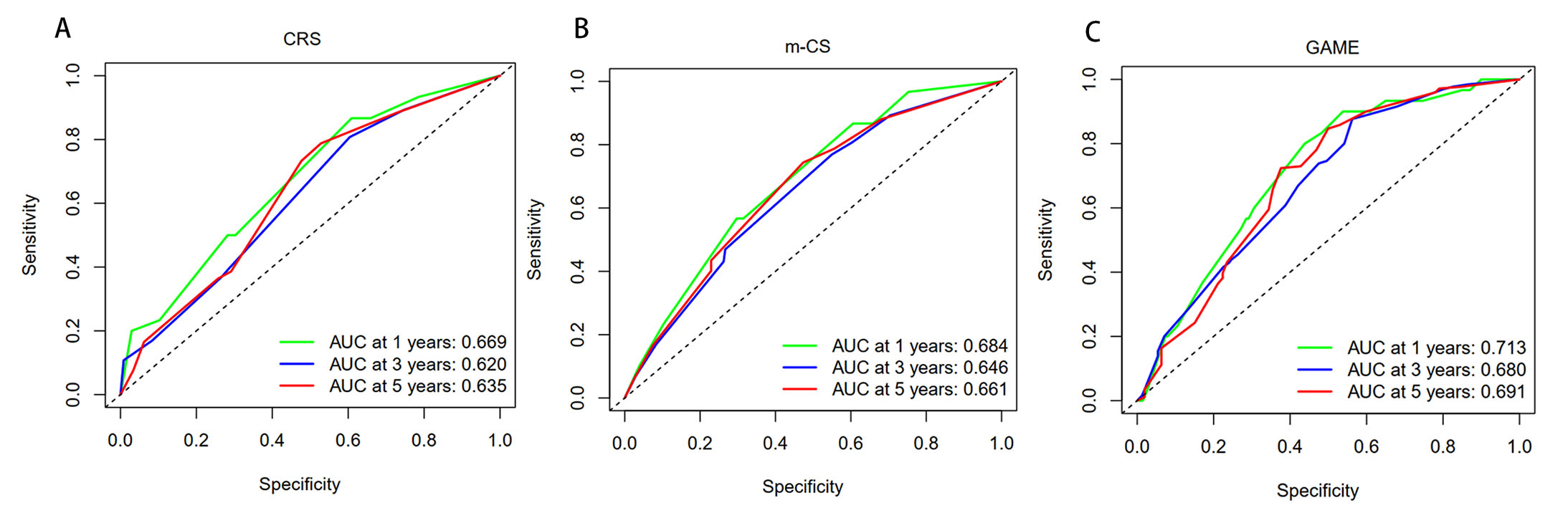

Supplement: Supplementary file 1 — Figure S1. [file CAM4-13-e7222-s002.jpg]

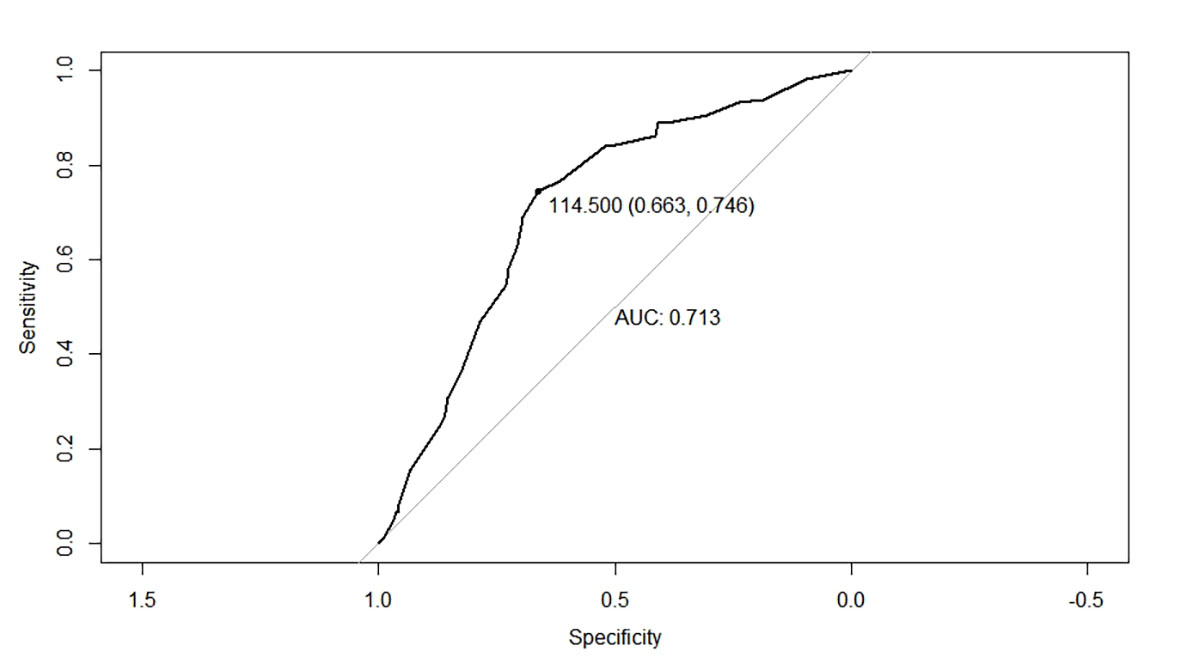

Supplement: Supplementary file 2 — Figure S2. [file CAM4-13-e7222-s001.jpg]
